# *Borrelia burgdorferi* Manipulates Innate and Adaptive Immunity to Establish Persistence in Rodent Reservoir Hosts

**DOI:** 10.3389/fimmu.2017.00116

**Published:** 2017-02-20

**Authors:** Karen E. Tracy, Nicole Baumgarth

**Affiliations:** ^1^Graduate Group in Immunology, University of California Davis, Davis, CA, USA; ^2^Center for Comparative Medicine, University of California Davis, Davis, CA, USA; ^3^Department of Pathology, Microbiology and Immunology, University of California Davis, Davis, CA, USA

**Keywords:** immune evasion, immune exhaustion, germinal center, complement inhibition, persistent infection, lyme disease

## Abstract

*Borrelia burgdorferi* sensu lato species complex is capable of establishing persistent infections in a wide variety of species, particularly rodents. Infection is asymptomatic or mild in most reservoir host species, indicating successful co-evolution of the pathogen with its natural hosts. However, infected humans and other incidental hosts can develop Lyme disease, a serious inflammatory syndrome characterized by tissue inflammation of joints, heart, muscles, skin, and CNS. Although *B. burgdorferi* infection induces both innate and adaptive immune responses, they are ultimately ineffective in clearing the infection from reservoir hosts, leading to bacterial persistence. Here, we review some mechanisms by which *B. burgdorferi* evades the immune system of the rodent host, focusing in particular on the effects of innate immune mechanisms and recent findings suggesting that T-dependent B cell responses are subverted during infection. A better understanding of the mechanisms causing persistence in rodents may help to increase our understanding of the pathogenesis of Lyme disease and ultimately aid in the development of therapies that support effective clearance of the bacterial infection by the host’s immune system.

## Introduction

*Borrelia burgdorferi* sensu lato is a species complex of spirochetal bacteria that infects a wide variety of mammals, birds, and reptiles. It includes, most notably, *Borrelia burgdorferi* sensu stricto (*Borrelia burgdorferi*), *Borrelia afzelii*, and *Borrelia garinii* (1), the three most prevalent causative agents of Lyme disease in humans (2). According to CDC reports, Lyme disease caused by *B. burgdorferi* is currently the most common vector-borne disease in the United States (3). In Europe, infections with *B. afzelii* and *B. garinii* are more prevalent than those with *B. burgdorferi*. In Asia, of the three primary disease-causing species, only *B. afzelii* and *B. garinii* are present (4). Other *Borrelia* species, including *Borrelia bavariensis* and *Borrelia spielmanii*, can also cause infection and disease in humans (5). Thus, *B. burgdorferi* sensu lato infections are an important global public health concern.

The bacteria are transmitted between hosts by ticks of the genus *Ixodes*. *B. burgdorferi* infection is therefore only common in areas where these vector species thrive. Deer mice (*Peromyscus leucopus*) are often considered the major reservoir host in the United States (6). In mice, spirochetes form persistent, non-resolving infections (7). However, these infections do not cause noticeable manifestations of disease in *P. leucopus* or certain common laboratory strains of mice [*Mus musculus*] (8–10). This suggests that *B. burgdorferi* has developed immune evasion strategies that allow it to persist in the face of a mammalian immune system. Such mechanisms may be the products of co-evolution with reservoir hosts, minimizing host disease manifestations while maximizing bacterial growth and transmission.

Laboratory mouse studies have been used to better understand *B. burgdorferi* pathogenesis in humans (11), just as they have for many other disease processes. They also provide an opportunity for better understanding the amplification and spread of *B. burgdorferi* in wild rodents, which in turn affects the infection risk of humans in endemic areas. Furthermore, *B. burgdorferi* in the mouse is an excellent model system for better understanding the mechanisms by which certain pathogens can achieve persistence in immunocompetent hosts (12, 13). Here, we summarize known mechanisms by which *B. burgdorferi* circumvents innate and adaptive immune responses to establish lifelong persistence in the mouse host. We examine interference with both the innate and adaptive immune systems. We emphasize bacterial persistence over disease and tissue pathology, because persistence of *B. burgdorferi* in wild rodents is a prerequisite for human infections.

## Evidence for Co-Evolution of *B. burgdorferi* with Vertebrate Hosts and Variation in Host Responses

A reservoir host is one in which a particular pathogen can survive with minimal affect to that host. The pathogen can subsequently be transmitted to other species that may experience ill effects. Incidental, or “dead-end,” hosts do not facilitate transmission of the pathogen to another host, but often experience manifestations of disease. This is the case for *B. burgdorferi*, where the reservoir hosts, including wild rodents [*Peromyscus* spp. (14, 15)] and passerine birds [canary finches (16)], are largely asymptomatically infected, whereas incidental hosts like humans can sometimes suffer a severe array of diseases, including arthritis, carditis, and skin and neurological disease manifestations (11, 17).

As outlined earlier, *Borrelia* species require both an invertebrate vector (ticks of the *Ixodes* genus) and a vertebrate host to complete their life cycle (11). Many ecological and evolutionary factors affect prevalence, persistence, and disease development by *Borrelia* infections in both vector and host. These factors include population dynamics, dispersal/migration, and evolution of all three players, as well as environmental landscape and climate (4). Further complicating the situation is the fact that both spirochetes (*B. burgdorferi* sensu lato) and the vectors (*Ixodes ricinus* species complex) are part of large species complexes, which have their own unique evolutionary patterns (4). Therefore, individual effects on *Borrelia* persistence can be difficult to untangle.

The existence of co-evolution with reservoir, but not incidental hosts like humans, remains to be rigorously tested with population genomics approaches (18). However, current evidence supports this hypothesis. For example, differential resistance to complement, an important and evolutionary conserved innate immune defense mechanism, has been suggested to drive host specializations of various *Borrelia* species to mammals, birds, and reptiles (19). In addition, there is evidence that selection acts on *B. burgdorferi*, within the reservoir host, to generate sequence diversity and polymorphisms relevant to virulence (20).

Co-evolution of host and pathogen must achieve a balance between the induction of immune mechanisms that reduce pathogen burden and pathogen-induced diseases, without clearing the infection. How this is achieved is incompletely understood. The continued presence of the pathogen in its host should provide ongoing triggers for both innate and adaptive immune response induction. Current research suggests that the immune system has important immune checkpoints that regulate immune responses, leading to a state of “immune exhaustion” during chronic infections. The process of immune exhaustion was first identified in chronic LCMV infection (21), leading to the discovery of distinct functions manifested in distinct transcriptional profiles of “exhausted T cells” in both mice and humans in response to a variety of chronic infections (22–25). The state of immune exhaustion goes beyond suppression of the T cell compartment alone, encompassing alterations in both innate and adaptive immune responses [reviewed in Ref. (26)]. While immune exhaustion has not been studied in the context of persistent infection with *B. burgdorferi*, it is conceivable that this process is also involved in the establishment of *Borrelia* persistence in its natural reservoir host. Indeed, mounting evidence suggest that the adaptive immune response is suppressed during *B. burgdorferi* infection.

## Evidence of *B. burgdorferi* Persistence

**Figure 1:**
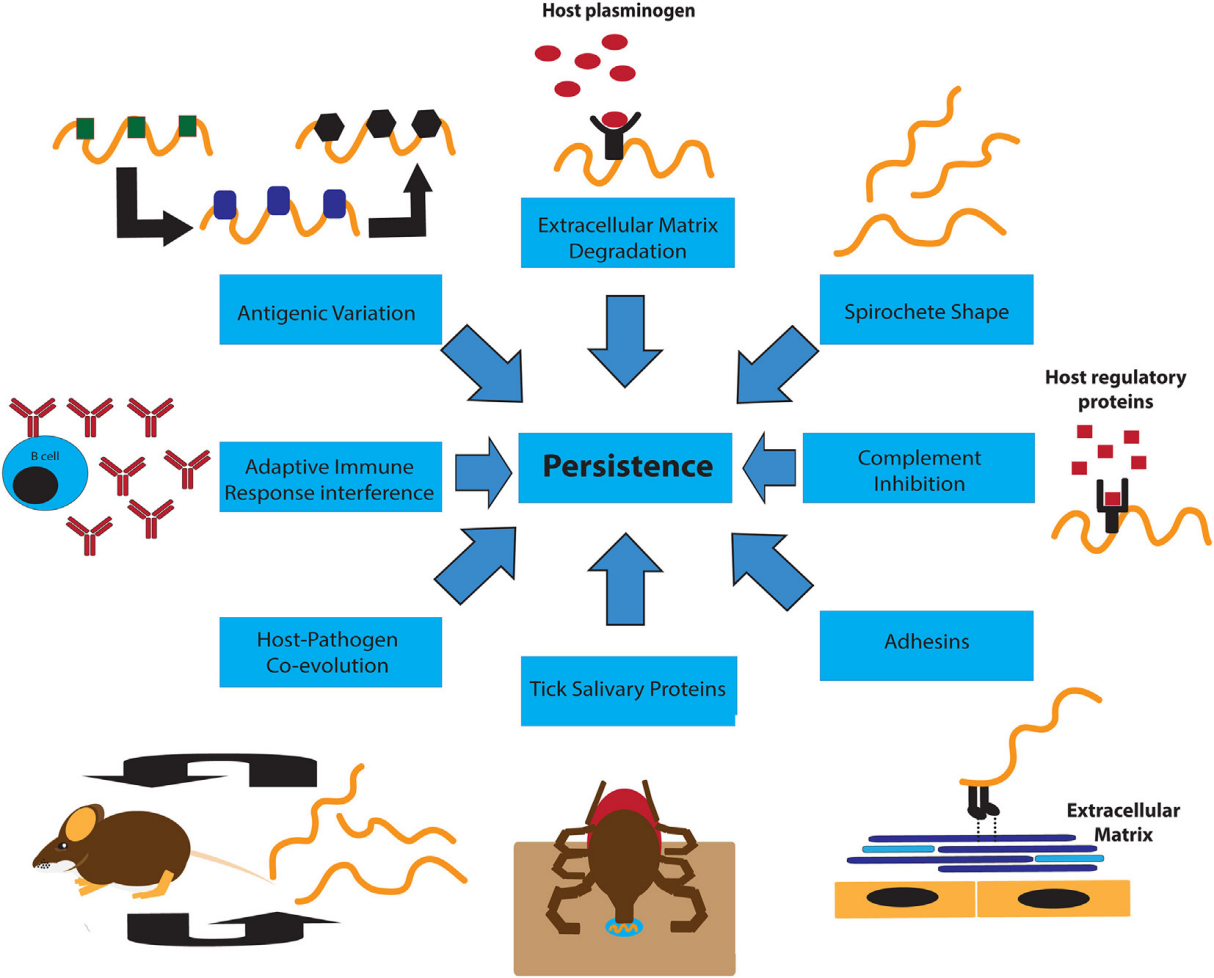
**An overview diagram of factors contributing to persistence of *Borrelia burgdorferi* in rodent hosts**. Shown are eight bacterial characteristics and mechanisms that *B. burgdorferi* may use to establish persistence in the rodent host: Spirochete shape (38, 123), antigenic variation and changes in gene expression (77, 83, 89), plasminogen binding and destruction of the extracellular matrix (52), interference with the adaptive immune response (69, 74, 75), host–pathogen co-evolution (20, 124), tick salivary protein-mediated immunosuppression (34), adhesins allowing entrance into the vasculature and tissues (49, 50), and interference with complement *via* CRASPs, and BBK32 (45, 60, 62, 64).

Persistent infection of reservoir hosts increases the odds that *B. burgdorferi* will be passed on to new hosts. It is challenging to distinguish between high prevalence of reinfection and/or true persistence occurring in the natural habitat of a reservoir host, such as the white-footed mouse *P. leucopus* (14, 15, 27). Experimental infections of *P. leucopus* have confirmed, however, that persistence of *B. burgdorferi* can occur, at least in laboratory settings (20). Experimental evidence also shows that *B. burgdorferi* persists in various laboratory mouse strains, either with or without disease manifestations, depending on the strain used (7, 8). Persistence after experimental infection was also observed in chipmunks (28), dogs (29), canaries (16), and non-human primates (30, 31). Figure 1 summarizes mechanisms that can support Borrelia persistence.

## Interference of *B. burgdorferi* with the Innate Immune System

Ticks provide the first defenses for *B. burgdorferi* against the innate immune system of the mammalian host. *B. burgdorferi* is transmitted from the tick to the mammalian host within <16–72 h after onset of tick feeding (32), where it encounters host defenses present in the skin. Tick salivary proteins play a role in suppressing the host immune system as long as the tick vector is attached. This includes the inhibition of vigorous activation of skin-resident macrophages and dendritic cells, including the suppression of cytokine and chemokine production, and inhibition of granulocyte recruitment to the site of the tick bite [reviewed in Ref. (33, 34)]. Multiple tick salivary proteins are known to interfere with the activation of the alternative complement pathway, potentially further supporting pathogen transmission [reviewed in Ref. (33)]. Mast cells also appear to be direct targets of salivary protein-mediated immune suppression (35). Inhibition of mast cells was facilitated through secretion of the salivary protein sialostatin L, which was shown to inhibit the induction of IL-9 production in the skin. IL-9 is known as an important regulator of pathogen-induced immune responses (35–37).

The morphology of spirochetes facilitates versatile motility that is predicted to play a role in the dissemination and persistence of *Leptospira, Treponema*, and *Borrelia* genera (38, 39). *B. burgdorferi* itself also expresses known immunomodulatory surface proteins, which help to modulate immune responses of the host. These proteins, specifically lipoproteins, have been studied extensively, and their wide variety of functions is reviewed elsewhere (40). We focus here on known functions of some proteins that are likely to contribute to persistence of *B. burgdorferi* in the rodent host (Table 1).

**Table 1:** ***B. burgdorferi*’s interface with mammalian hosts and its effect on *Borrelia* survival**.

Immune response modulators	Effects	Reference
Co-evolution of *B. burgdorferi* and its hosts	Host specialization and evolution of virulence- and infectivity-associated genes	(19, 20)

Tick salivary proteins	Suppression of pro-inflammatory responses in the host	(33, 34, 125)

Spirochete morphology and motility	Increase in *B. burgdorferi* dissemination and persistence	(38, 39)

*B. burgdorferi* adhesins	Interactions with host tissues, contributing to dissemination and persistence	(49, 50)

Host interactive proteins	Binding to host enzymes, such as plasmin/plasminogen; facilitates extracellular matrix degradation	(50, 52)

CRASPs	Decreased and inhibited complement activation	(60, 62, 63)

Modulation of protein expression	Adaptation to host, downregulation of immunogenic proteins, and antigenic variation	(79, 80)

Inappropriate macrophage activation	Extracellular matrix degradation	(54)

Antibodies with IgM-skewed isotype profile and of low affinity	Decreased antibody response quality which may contribute to persistence	(see text footnote) (73–75)

Loss of demarcated T and B cell zones in secondary lymphoid tissues and collapse of germinal centers	Reduced antibody class switch recombination and somatic affinity maturation. Failure to induce long-lived plasma cells and memory B cells in a timely manner	(69, 73, 74, 110)

One important group of such proteins is the adhesins, which mimic host integrins, molecules that facilitate attachment and migration. Thus, *B. burgdorferi* seems to subvert existing mechanisms regulating immune cell migration for its benefit. Adhesins are an important category of bacterial virulence factors that protect bacteria from clearance by physical forces such as mucociliary clearance, facilitate homing to and entrance into host tissues that act as important pathogen niches and trigger signaling events in host cells (41). BBK32 is one such adhesin. At the initial site of infection, BBK32 was shown to create “catch bonds” that slow bacterial movement enough for flagella-driven entrance of *B. burgdorferi* into the vasculature (42). This helped to explain why bacterial loads in the blood are higher when BBK32 is expressed (43). Once in the blood, BBK32 seems to help *B. burgdorferi* to target joint tissues for colonization *via* binding to glycosaminoglycan (44). However, recent studies suggested that BBK32 has additional, complement-inhibitory, functions (45). Specifically, it was found to bind to the C1 complex and thereby to inhibit the classical pathway of complement activation, i.e., antibody-mediated bacterial clearance (45).

Another adhesion protein of *B. burgdorferi*, p66, was shown to interact with ligands on the host vasculature to facilitate *B. burgdorferi* extravasation from blood vessels into tissues (46). *B. burgdorferi* also expresses two decorin-binding proteins (Dbp), DbpA and DbpB. Surface expression of these proteins seems to increase the level of tissue colonization (47). They seem to support persistence of *B. burgdorferi* in tissues that express high levels of decorin (joint tissue and skin) (48). Further details about *B. burgdorferi*-expressed adhesins have been summarized in recent reviews (49, 50).

Once bacteria have gained access to a specific organ or tissue, they require proteases that degrade the extracellular matrix (ECM), enabling the bacteria to move between cells deeper into the tissues. This is particularly important for *B. burgdorferi*, which targets ECM-rich connective tissues (50, 51). The genome of *B. burgdorferi* does not seem to contain any known ECM-degrading proteases. Instead *B. burgdorferi* is able to bind host urokinase and plasminogen, a multifunctional serum protein that can initiate ECM and fibrinogen degradation (52). Plasminogen can also inhibit complement activation and promote complement degradation (53).

Furthermore, *B. burgdorferi* can induce host cells such as macrophages to secrete matrix metalloproteases (MMPs), particularly the gelatinase MMP-9, *via* TLR2-mediated immune activation (54). MMP-9 was shown to be selectively upregulated in erythema migrans skin lesions during acute *B. burgdorferi* infections of humans (55) and is thought to help *B. burgdorferi* tissue dissemination by enabling the degradation of the ECM. However, studies by Hu and colleagues recently demonstrated that MMP-9 expression is not required for *B. burgdorferi* dissemination. Instead, it regulated the influx of inflammatory cells, and thereby Lyme arthritis, indirectly by the degradation of collagen in joints (56). Plasminogen-binding proteins have not been shown to increase *B. burgdorferi* persistence *per se*, but they do facilitate the entrance of *B. burgdorferi* into the ECM of tissues, where bacteria maybe protected from innate immune response mechanisms such as complement-mediated degradation and/or neutralization by early-induced IgM antibodies.^1^

### Interference of *B. burgdorferi* with the Complement System

As mentioned earlier, inhibition of the complement system is an important immune evasion strategy employed by many pathogens, including *B. burgdorferi* (57–59). *B. burgdorferi* proteins that interfere with complement activation allow for survival and dissemination of the pathogen from the initial site of infection (60).

The complement system consists of an evolutionarily highly conserved family of proteins that are found in all body fluids and serve three main functions during infection: trigger inflammation, opsonize pathogens, and form the “membrane attack complex” (formation of a pore in the cell membrane that causes cell lysis). Classical, alternative, and lectin are the three distinct pathways by which complement-mediated signaling and bacterial killing can be initiated [described in more detail in Ref. (61)]. Independent of the initial trigger, all pathways lead to the formation of a protease, the C3 convertase, which cleaves the complement component C3 into its activated components C3a (an inflammatory mediator) and C3b (an opsonin and immune stimulatory protein). The complement component C3b can also form the C5 convertase, another protease that cleaves complement component C5 into C5a and C5b, leading to the formation of the bactericidal “membrane attack complex” (61). C5a, like C3a, is a strong inducer of inflammation. During the process of complement activation, another complement component, C4, is cleaved into C4a and C4b. The latter also acts as an opsonin. Because of these multiple and highly pro-inflammatory effects, systemic activation of complement can cause septic shock if not appropriately regulated. Regulators of overshooting complement activation include components of the complement system itself: the Factor H family proteins and the C4b-binding protein. These proteins inhibit complement activation by a variety of mechanisms, including by accelerating the decay of C3 convertases, thus interrupting the complement activation cascade.

*Borrelia burgdorferi* has evolved complex mechanisms to evade complement-mediated killing by binding to the inhibitory host-regulatory factors [reviewed in Ref. (60, 62)]. *B. burgdorferi* expresses a diverse family of complement regulator-acquiring surface proteins, which recruit Factor H family proteins [reviewed in Ref. (63)]. Factor H and its relatives primarily inhibit activation of the alternative complement pathway. More recently, it was discovered that *B. burgdorferi* also binds to host C4-binding proteins, which primarily inhibit activation of the classical and lectin pathways (64). And, as outlined earlier, BBK32 seems to inhibit the classical pathway of complement activation *via* binding to the C1 complex (45). Thus, *B. burgdorferi* seems to target all three activation pathways of the complement cascade. The effects of complement inhibition on adaptive immune responses are outlined below.

## Interference of *B. burgdorferi* with the Induction of Adaptive Humoral Immune Responses

High levels of *B. burgdorferi* antigen-specific antibodies are produced during infection, and they have the capacity to prevent reinfection with the same *B. burgdorferi* strains (65–67). The antibody response also results in reduction, but not elimination, of *B. burgdorferi* from tissues (68). Both T-independent and T-dependent antigens are targeted by the humoral immune response, representing a wide variety of surface proteins with different functions (20, 67, 69). How then does *B. burgdorferi* avoid antibody-mediated clearance?

### *B. burgdorferi*-Induced Humoral Immunity

The strong production of *B. burgdorferi* antigen-specific antibodies, as well as the strong increases in *B. burgdorferi* tissue load in SCID mice and B cell-deficient mice compared to wild-type controls (70, 71), has long been considered evidence of a robust and effective B cell response against *B. burgdorferi* infection. However, while the data provide clear evidence that B cells play an important role in the control of *B. burgdorferi* infection, this does not mean that these responses are optimally induced. Considering that *B. burgdorferi* infection results in persistent infections of mice despite these robust antibody responses, it is important to also consider the *quality* of the *B. burgdorferi*-specific antibody response. Several factors are known to affect the efficacy of humoral immune responses, including the epitope specificity of the induced antibodies, their immunoglobulin isotype profile, binding avidity to their cognate antigens, and various posttranslational modifications that can affect their effector functions (6, 20).

IgM is the first antibody isotype secreted during an immune response. It is important in controlling bacteremia and in activating the classical complement pathway. Immunoglobulin class switch recombination (CSR) to IgG typically occurs soon after an infection, and the four subtypes of murine IgG work together effectively to clear most pathogens (72). However, during *B. burgdorferi* infection, serum IgM levels remain high throughout infection (see text footnote). Moreover, hybridomas generated from lymph nodes of mice on days 8 and 18 postinfection showed that nearly half of *B. burgdorferi* antigen-specific cells were producing IgM, and the ratio of IgM to IgG never significantly changed throughout the infection (see text footnote) (73). The strong and ongoing production of IgM cannot be explained entirely by a predominance of T-independent responses, because depletion of CD4 T cells decreased the number of IgM-antibody-secreting cells (ASCs) (74). Thus, infection with *B. burgdorferi* induces an antibody response that is characterized by the continued production of IgM and IgG. Further studies will need to determine whether the strong production of IgM is evidence of a strong beneficial immune response or whether *B. burgdorferi* might be subverting the B cell response to generate this unusual Ig-isotype profile. Our studies have failed to find any beneficial effect of IgM on control of bacterial dissemination or *B. burgdorferi* tissue loads (see text footnote).

Given the important role of T cells in the regulation of CSR by B cells, the data may indicate a deficiency in T helper cell activation and/or functionality. *In vitro* data indeed provided some evidence that the CD4 T helper cell response induced by *B. burgdorferi* infection is distinct in function from that of CD4 T cells induced by immunizing mice with inactivated *B. burgdorferi* (75). T-dependent B cell responses usually also result in significant affinity maturation, i.e., an increase in the binding avidity of antibodies to their cognate antigens over time. Measuring antibody avidities of serum antibodies to a representative T-dependent antigen on *B. burgdorferi* N40, arthritis-related protein (Arp), however, failed to provide evidence for affinity maturation in the serum response to *B. burgdorferi* (75). Instead, we found that the binding avidity of the serum antibodies to Arp initially increased for the first 6 weeks of infection, but then peaked and steadily decreased thereafter to levels seen at the beginning of the infection. The rate of drop in antibody affinity was consistent with the normal half-life and decays kinetics of serum antibodies (75), suggesting that the ASC that generated the higher-affinity antibodies were short-lived.

Both CSR and hyperaffinity maturation of antibodies are T-dependent processes that usually occur in germinal center (GC) reactions in secondary lymphoid tissues. As we will outline below in more detail, we noted a collapse of the GC responses that coincided with the peak and then reversal of the antibody avidities. The data strongly suggest that the T-dependent GC responses are not fully functional during *B. burgdorferi* infection. The fact that immunization with inactivated *B. burgdorferi* infection resulted in robust GC responses suggests that exposure to live *B. burgdorferi* results in a subversion of the B cell response (73, 74). On the basis of these data, we propose that although present in large quantities, the B cell responses to and functionality of the serum antibody response to *B. burgdorferi* are suboptimal, enabling *B. burgdorferi* persistence, while also controlling *B. burgdorferi* tissue loads and thus overwhelming infection. Alterations in the B cell response quality and/or antibody functionality could provide a powerful immune evasion strategy for bacteria that are clearly susceptible to antibody-mediated immune clearance mechanisms (65, 66, 70, 71, 76, 77).

### Modification of *B. burgdorferi* Outer Surface Protein Expression

Although *B. burgdorferi* surface proteins have many functions in evading recognition by the host immune system, they are also antigens that trigger antibody responses. Additional immune evasion strategies therefore likely exist that inhibit recognition and antibody-mediated clearance of *B. burgdorferi*.

*Borrelia burgdorferi* is known to undergo major protein expression changes over the course of its life cycle, including expression of outer surface proteins. During transmission from the tick to a mammalian host, environmental signals trigger extensive global changes in gene expression (78, 79). Changes in surface protein expression also occur within a mammalian host during the course of infection. At least some proteins that trigger strong antibody responses are downregulated during the chronic phase of infection (80). One important example is OspC, a T-dependent antigen that is essential for initial colonization of mammalian hosts. Shortly after infection, this lipoprotein is no longer required, and if not downregulated triggers a strong and effective antibody response. However, expression is usually rapidly lost upon infection (81–84). This appears to be an effective immune evasion strategy, because constitutive expression of OspC prevents spirochete persistence, whereas bacteria that successfully downregulate OspC outcompete expressors *in vivo* (76, 85).

Antigenic variation is a process by which a pathogen varies the sequence of an expressed protein to avoid the deleterious effects of antibodies raised against it. Antibody-target switching has been demonstrated as an effective immune evasion strategy for numerous pathogens, including the closely related relapsing fever pathogen *Borrelia hermsii* (86–88). The *B. burgdorferi* genome contains the variable surface antigen E (*vlsE)* locus, which has received much attention as a major immunodominant surface protein *of B. burgdorferi* that undergoes extensive and rapid antigenic variation in mammalian hosts [reviewed in Ref. (89)]. In *B. burgdorferi* strain B31, VlsE recombination seems to be critical for *B. burgdorferi* persistence and the ability of *B. burgdorferi* to reinfect a host following antibiotic treatment (77, 90–92). It is interesting to note that vlsE recombination also occurs in antibody-deficient SCID mice, but not *in vitro*, suggesting that host triggers other than the antibody response direct this process (93). While it seems that a lack of variation of vlsE results in the rapid clearance of *B. burgdorferi*, it is less clear whether this process alone can explain the effective evasion of *B. burgdorferi* from antibody-mediated clearance. Recently, mathematical models have been put forward to suggest that a strongly immunodominant variable surface protein may prolong immune responses long enough to drive immune exhaustion (94), an intriguing idea that requires testing in the context of *B. burgdorferi* infection.

Finally, extensive genetic variation exists between the various *Borrelia* species and between individual bacteria (95–98). This allows adaptation to selective pressure from the host immune system, as well as to larger ecological niches, allowing strains to become fitter in a given geographic area (95). It also applies during the course of a chronic infection, as bacterial variants compete with each other (85, 99). Variation between strains is large enough in the context of the antibody response that different strains can infect the same mouse (100). Reinfection with the same strain has been tested in experimental settings after antibiotic treatment of mice. Studies by Piesman et al. and by Elsner et al. suggested that antibody-mediated protection wanes over time, even to the same strain of *B. burgdorferi* (69, 101). Given that long-term antibody production is usually induced following an infection, facilitated by the development of long-lived plasma cells that migrate to and then reside in the bone marrow (102, 103), the data further suggest that the antibody response to *B. burgdorferi* infection lacks some of these key components of successful humoral responses. It can be expected that each of the above outlined immune evasion mechanisms is unlikely to be solely responsible for persistence; instead they may act in concert. Whether these multiple apparent strategies represent redundant or synergistic effects remains to be established.

### *B. burgdorferi* Modulates the Adaptive Immune Response

Modeling of population dynamics supports the establishment of equilibrium between *B. burgdorferi* and the host’s adaptive immune response (104). This is consistent with recent findings suggesting that the humoral immune response and, more specifically, the T-dependent B cell responses are a particular target of *B. burgdorferi*-induced immune suppression.

After escaping the site of infection, *B. burgdorferi* disseminates to other tissues *via* blood and lymph (43, 105). Mechanisms of entrance into the blood are better studied, but live *B. burgdorferi* is also capable of entering, persisting in, and traveling through the lymphatics (73, 105). Dissemination *via* the lymphatics has been described in other infections as an effective technique for immune evasion due to the specialized cell populations and altered immune milieu in these vessels compared to the bloodstream (106–108). Shortly after infection with cultured or host-adapted spirochetes (HAS), live *B. burgdorferi* enter lymph nodes draining the site of infection, resulting in massive lymph node enlargement (73, 109).

In secondary lymphoid tissues such as the lymph nodes, naïve T cells encounter antigens for the first time, and activated antigen-specific B cells receive the signals they need to proliferate. During *B. burgdorferi* infection, the presence of live spirochetes seems to interfere with these processes. Between days 5 and 7 postinfection of C57BL/6 mice with HAS, the separation of T cell zones and B cell follicles completely was lost within the draining lymph nodes. This degradation was not seen in mice that were inoculated with heat-inactivated spirochetes, suggesting that an active bacterial process causes the disruption of the normal immune response of the host (73, 74, 110). Interestingly, similar observations have been made also following infections with other pathogens, including infections with *Plasmodium* and *Salmonella* (111, 112). While during *Salmonella* infection lymph node architecture disruption was dependent on TLR4-mediated signaling (112), the observed changes during *B. burgdorferi* infection were independent of MyD88 and TRIF signaling (110). Given the importance of T and B cell trafficking within the lymph nodes for initiating and maintaining T–B interaction and immune response regulation, the data suggest that multiple pathogens, including *B. burgdorferi*, may have evolved strategies to interfere with the earliest processes of adaptive immune induction.

Furthermore, the presence of live (but not heat-killed) *B. burgdorferi* caused a disproportionate recruitment and proliferation of naïve follicular B cells, but not CD4 T cells, to the affected lymph nodes. This appeared to be a separately controlled process, but one also mediated specifically by live bacteria. B cell recruitment and/or retention required signaling through the type I interferon receptor by non-hematopoietic cells, likely lymph node stromal cell compartments (110). The resulting accumulation of large numbers of B cells in the lymph nodes explains the lymphadenopathy that is also observed in infected humans and dogs (113, 114). This pattern of lymph node architecture disruption followed by influx/retention of B cells is recapitulated later in more distant lymph nodes as they are infiltrated by the migrating spirochetes (73, 110).

The data support the concept that *B. burgdorferi* interferes with the induction and maintenance of adaptive immune responses by altering or hijacking innate immune signaling pathways. What effect does this have on persistence of *B. burgdorferi*? The organized lymph node structure and the regulated migration of lymphocytes within secondary lymphoid tissues is crucial for effective interaction of T and B cells and the induction of adaptive immune effector mechanisms. Disruption of this structure is liable to have long-term negative consequences for the quality of the immune response and thus increases the likelihood of pathogen persistence. We outline below evidence to this effect.

### Interference of *B. burgdorferi* with GC Formation and Maintenance

Activated B cells in the draining lymph nodes proliferate rapidly and initially form structures called extrafollicular foci (73). In extrafollicular foci, B cells become plasmablasts with or without T cell help and produce large quantities of *B. burgdorferi*-specific antibodies rapidly. B cells in extrafollicular foci, however, do not undergo extensive affinity maturation, nor do they form memory B cells or long-lived plasma cells (115). For this, GCs are needed. GCs are a complex and highly organized structures that form within the B cell follicles of secondary lymphoid tissues that consist of GC B cells, CD4 T follicular helper cells (T_FH_), and follicular dendritic cells (FDCs). B cell activation and proliferation in GCs is facilitated by interaction with CD4 T cells and presentation of antigens on FDCs. High-frequency insertion of random mutations into the antigen-binding variable domain of the antibody molecule during this process, and the competition of the newly generated B cells for binding to antigen and for T cell-help, is thought to drive B cells with the highest affinity for antigen to outcompete other clones with weaker antigen binding. Although mechanisms are still unclear, GC responses lead to the development of two distinct cell populations: memory B cells, i.e., non-ASCs that circulate and can respond vigorously to repeat infection, and long-lived plasma cells, which continuously produce antibodies from their bone marrow niches and contribute to immune protection from reinfections (116–119). The GC environment also promotes CSR to IgG.

Despite the early presence of *B. burgdorferi* in the draining lymph nodes, GC B cell and T_FH_ numbers remain low for the first 2 weeks in experimentally infected mice (74) and then become measurable. However, although live *B. burgdorferi* remained present in the lymph node for at least 1 year after infection, and thus, *B. burgdorferi* antigens were presumably continuously available, the GCs collapsed around 1 month after infection, and associated B and T cell numbers decreased steadily over the next month (69). As outlined earlier, the decline in the avidity of serum antibodies against Arp follows the collapse of GCs (69).

Consistent with the short-lived nature of the GC responses, their functional ineffectiveness was demonstrated by experiments showing a complete lack of memory recall responses to both *B. burgdorferi* antigens and a co-administered vaccine antigen for many months after infection with *B. burgdorferi*. Stable continued antibody production by long-lived ASCs in the bone marrow was also strongly delayed for at least 3 months after infection (69, 74). The delay in these important B cell response outcomes is especially dramatic considering a mouse’s relatively short life span and likely frequent exposure in the wild. Although mice do not clear infection with *B. burgdorferi*, impairing the memory response could be advantageous to *B. burgdorferi* by leaving the host susceptible to secondary or superinfections. It might also prevent a timely and strong response to antigens that are dynamically upregulated and downregulated by *B. burgdorferi* as the infection progresses.

By decreasing the capacity of the host to produce effective antibodies against *B. burgdorferi*, the GC collapse may help *B. burgdorferi* evade clearance. The signals and mechanisms leading to the collapse, however, are unknown. One possible mechanism is the interference of *B. burgdorferi* with the complement system. Continued antigen presentation is crucial for hyperaffinity maturation, and components of the complement system are known to be involved in this process. Specifically, activated C3 and C4 fragments bound to antigen and adhere to complement receptors 1 and 2 (CR1 and CR2). These receptors are present on the major antigen-presenting cells in the GC, the FDC, and on GC B cells. It was shown previously that GCs will form normally in mice lacking CR1 and CR2, but collapse prematurely, before GCs can perform their important functions (120). This phenotype is strikingly similar to that seen in wild-type mice infected with *B. burgdorferi*. Interestingly, in *B. burgdorferi*-infected mice, although CR1 and CR2 are present on FDCs and GC B cells, C4 is not detectable (69). C4 is typically deposited on the surface of FDCs supporting antigen presentation. Interference with C4 deposition could inhibit antigen presentation by FDCs to GC B cells and thereby lead to GC collapse. *B. burgdorferi* interference with activation of complement could also have various indirect effects on GCs: changing the cytokine milieu, reducing antigen presentation to naïve B cells *via* CR1 on APCs outside the GC, reducing naïve B cell activation *via* co-stimulation with CR2, and reducing opsonization (and thus uptake) of antigens. Exploring the role of complement and complement inhibition by *B. burgdorferi* during infection are important subjects for future studies.

## Conclusion

The phenomena described earlier represent potential novel mechanism(s) for manipulation of the adaptive immune system by a pathogen that establishes persistent infections in its reservoir host. Elucidation of these mechanisms has important translational and clinical applications. A better understanding of how *B. burgdorferi* persists long term in rodents would be useful for understanding public health risks and devising appropriate preventative measures in endemic areas. Given the extensive similarities in the immune system of rodents and humans, it seems likely that the mechanisms of immune evasion and suppression outlined here may also be active in at least some infected humans. The induction of diseases such as carditis, arthritis, acrodermatitis chronica atrophicans, and neuroborreliosis seen in some patients with Lyme disease and infected companion animals, but rarely in mice, suggest maladaptation of *B. burgdorferi* to these hosts. Humans developing these inflammatory diseases to *B. burgdorferi* infection may have an immune system that is ineffectively suppressed by *B. burgdorferi*. There is good experimental evidence that a block of pro-inflammatory T cell responses, such as facilitated through blockade of IL-12, will cause reductions in arthritis development in C3H mice, but it also causes increases in *Borrelia* tissue loads (121, 122). Development of therapeutics that can shift the balance toward immune activation and bacterial clearance without causing inflammation-induced diseases might provide superior tools to the current antibiotic therapies.

Much remains to be elucidated about the mechanisms by which *Borrelia* evades the host response. This area of research provides a particularly rich ground for collaboration among evolutionary biologists, ecologists, microbiologists, and immunologists.

## Author Contributions

KT and NB conceived of and wrote the article.

## Conflict of Interest Statement

The authors declare that the research was conducted in the absence of any commercial or financial relationships that could be construed as a potential conflict of interest.

## Footnotes

^1^Hastey CJ, Elsner RA, Olsen KJ, Tunev SS, Escobar ED, Barthold SW, et al. *Borrelia burgdorferi* infection-induced IgM controls bacteremia but not bacterial dissemination or tissue burden. (submitted).
